# Anti-β2GPI IgG display a broad reactivity against different β2GPI domains beyond domain 1: results from the APS ACTION and multi-center Italian cohorts

**DOI:** 10.3389/fimmu.2026.1809192

**Published:** 2026-05-01

**Authors:** Claudia Grossi, Caterina Bodio, Suresh Kumar, Elisa Bison, Ricard Cervera, Maria Gerosa, Michael Mahler, Diana Paredes-Ruiz, Silvia Piantoni, Massimo Radin, Savino Sciascia, Esther Rodriguez-Almaraz, Maria G. Tektonidou, Angela Tincani, Vittorio Pengo, Davide Soranna, Antonella Zambon, Maria Laura Bertolaccini, Hannah Cohen, Doruk Erkan, Nicola Pozzi, Francesco Tedesco, Maria Orietta Borghi, Pier Luigi Meroni

**Affiliations:** 1 Laboratory of Immuno-Rheumatology, IRCCS Istituto Auxologico Italiano, Milan, Italy; 2 Edward A. Doisy Department of Biochemistry and Molecular Biology, Saint Louis University School of Medicine, St. Louis, MO, United States; 3 Thrombosis Research Laboratory, Department of Cardio-Thoracic-Vascular Sciences and Public Health, University of Padova, Padova, Italy; 4 Department of Autoimmune Diseases, Hospital Clinic, University of Barcelona, Barcelona, Spain; 5 Dipartimento di Scienze Cliniche e di Comunità, Dipartimento di Eccellenza 2023-2027, University of Milan, Milan, Italy; 6 Rheumatology Clinic, ASST-G.Pini-CTO, Milan, Italy; 7 Research and Development, Headquarters & Technology Center Autoimmunity, Werfen, San Diego, CA, Italy; 8 Autoimmune Diseases Research Unit, Department of Internal Medicine, Biobizkaia Health Research Institute, Hospital Universitario, Cruces, Spain; 9 Rheumatology and Clinical Immunology Unit, ASST Spedali Civili, Department of Clinical and Experimental Sciences, University of Brescia, ERN-Reconnect Member, Brescia, Italy; 10 Department of Clinical and Biological Sciences, University Center of Excellence on Nephrologic, Rheumatologic and Rare Diseases (ERK-Net, ERN-Reconnect and RITAERN Member) with Nephrology and Dialysis Unit and Center of Immuno- Rheumatology and Rare Diseases (CMID), Coordinating Center of the Interregional Network for Rare Diseases of Piedmont and Aosta Valley, San Giovanni Bosco Hub Hospital, University of Turin, Turin, Italy; 11 Hospital Universitario 12 de Octubre, Madrid, Spain; 12 Rheumatology Unit, First Department of Propaedeutic Internal Medicine, Joint Academic Rheumatology Program, Medical School, National and Kapodistrian University of Athens, Athens, Greece; 13 Biostatistic Unit, IRCCS Istituto Auxologico Italiano, Milan, Italy; 14 Laboratory of Quantitative Methods for Life, Health and Society, Department of Statistics and Quantitative Methods, University of Milano-Bicocca, Milan, Italy; 15 Academic Department of Vascular Surgery, School of Cardiovascular and Metabolic Medicine & Sciences, King’s College, London, United Kingdom; 16 Department of Haematology, University College London, London, United Kingdom; 17 Barbara Volcker Center for Women and Rheumatic Diseases, Hospital for Special Surgery, Weill Cornell Medicine, New York, NY, United States

**Keywords:** antibodies, antiphospholipid syndrome, diagnostic assays, domains, β2GPI

## Abstract

**Background:**

Anti-β2glycoprotein I (β2GPI) antibodies are the hallmark of the antiphospholipid syndrome (APS). β2GPI consists of five domains, DI-DV. While DI is the primary target, the clinical relevance of antibodies against other domains remains uncertain. We analyzed two large anti-β2GPI IgG positive cohorts, to investigate whether different domain binding affects anti-β2GPI IgG assay testing and APS diagnosis.

**Methods:**

The presence of anti-DI and anti-DIV/DV antibodies (by chemiluminescence (CLIA) and in-house ELISA, respectively) was searched in an APS ACTION (n.191) and Italian validation (n.105) cohorts. In the latter, we detected anti-β2GPI IgG reactivity by four assays (in-house and commercial ELISA, CLIA, and fluorescence enzyme immunoassay), and used a modified anti-β2GPI IgG in-house ELISA with recombinant single-domain-lacking β2GPI variants to evaluate domain-dependent reactivity.

**Results:**

We confirmed anti-DI, anti-DIV/DV β2GPI IgG direct reactivity in classified and non-classifiable APS in the two cohorts, and found anti-DI/anti-DIV/DV double negative (38/191, 29/105) and anti-DIV/DV single-positive (6/191, 9/105) samples. Anti-β2GPI IgG discordant samples by the four methods had the highest presence of anti-DIV/DV single-positives and anti-DI/anti-DIV/DV double-negatives, compared to four-method concordant samples: 16% vs 2% and 45% vs 11% (p <0.0001), respectively. The single-domain molecule-based assay showed that the APS serum samples depended mainly on DI, DV, and DII, slightly on DIV, and not at all on DIII.

**Conclusions:**

Serum IgG from both classified and non-classifiable APS may react with other β2GPI domains than DI and DIV-V. Anti-β2GPI domain selectivity can explain discordant results among diagnostic assays.

## Introduction

Antibodies targeting β2-Glycoprotein I (β2GPI) play a central role in the pathogenesis of antiphospholipid syndrome (APS). Their ability to mediate the lupus anticoagulant (LA) phenomenon and to account for positive results in the anti-cardiolipin (aCL) assay underscores their diagnostic and prognostic relevance (1).

β2GPI (also known as Apolipoprotein H) is a plasma protein present at relatively high concentrations (~200 µg/ml). It is composed of five domains (DI–DV) and has a molecular weight of approximately 50 kDa (2, 3). The first four domains (DI–DIV) are canonical complement control protein modules, while the fifth domain (DV) is structurally distinct, containing an additional loop that enables interaction with anionic phospholipids (4–8), lipopolysaccharides (LPS) (8), and potentially various cell membrane receptors (9–11). Despite its well-established role in APS, the physiological function of β2GPI remains incompletely understood and, at times, controversial, particularly regarding its involvement in complement regulation (12, 13) and coagulation, where both anticoagulant and procoagulant roles have been proposed (3, 14–17).

Given its multidomain structure, β2GPI can be targeted by antibodies recognizing distinct epitopes. Robust evidence indicates that in patients with thrombotic/obstetric APS, anti-β2GPI antibodies are predominantly directed against DI (18–20). However, the prevalence and clinical relevance of antibodies targeting other domains remain unclear. While little is known about antibodies against DII and DIII, some data exist for DIV and DV (6, 7). Anecdotal reports have described polyclonal aPL targeting the DIV-DV fragment in both non- APS classifiable individuals and patients with vascular or obstetric APS, albeit at much lower titers than anti-DI antibodies (21–25). High titers or isolated anti-DIV/DV IgG have been observed in asymptomatic aPL-positive carriers and in a handful of patients with atypical APS presentations (26–29), leading us to propose a non-pathogenic role of these autoantibodies (30, 31). Yet, their prevalence and relationship with anti-DI antibodies in large multicenter cohorts remain to be established.

In this study, we examined two large multicenter cohorts of aPL-positive individuals to explore how IgG antibodies targeting specific β2GPI domains relate to different APS subtypes. We also evaluated whether these associations are consistent across various diagnostic platforms. Our findings confirm and extend previous data showing that both in classified and non-classifiable APS 1) β2GPI domains other than DI contribute to antibody reactivity towards the β2GPI whole molecule, 2) anti-DI/anti-DIV/DV double negative and anti-DIV/DV single-positive domain specificity exists, and 3) is responsible, at least partially, of discordancy among the four anti-β2GPI IgG assays we used for this work.

## Materials and methods

### Patients

The study involved two different cohorts of aPL-positive patients. The first cohort, the discovery cohort, comprises 291 patients recruited by eight APS ACTION centers (32). All the patients were positive for at least one of the APS laboratory classification criteria (LA, anti-β2GPI IgG/IgM, anti-Cardiolipin (aCL) IgG/IgM) at inclusion; aPL solid-phase assay profile was measured by QUANTA Flash^®^ β2GPI IgG chemiluminescence (CLIA) at APS ACTION core laboratories. The second cohort, the validation cohort, included 133 aPL-positive patients, enrolled at three Italian centers. Patients were classified as having APS according to the revised Sapporo classification criteria and fulfilled the 2023 ACR/EULAR classification (33, 34). Those who were positive for aPL but without APS were labeled as non-APS classifiable aPL positive subjects. Clinical, laboratory, and demographic characteristics of the two cohorts are presented in **Supplementary Tables 1S**, **2S**. Serum samples were collected and stored at -20 °C until testing. Samples were pseudo-anonymized. The study was approved by the Ethical Committee of the Istituto Auxologico Italiano (22072010) and conducted in accordance with the Declaration of Helsinki.

### Anti-β2GPI IgG antibody detection methods

Levels of anti-β2GPI antibodies in the discovery cohort were measured by QUANTA Flash^®^ β2GPI IgG CLIA (Inova Diagnostics, San Diego, CA, USA). In the validation cohort, we tested all the samples by four anti-β2GPI IgG methods: in-house ELISA, QUANTA Lite^®^ β2GPI IgG ELISA (Inova Diagnostics, San Diego, CA, USA), QUANTA Flash^®^ β2GPI IgG CLIA (Inova Diagnostics, San Diego, CA, USA), and EliA™ β2-Glycoprotein I IgG fluorescence enzyme immunoassay (FEIA; Thermo Fisher Scientific, Freiburg, Germany). The assays were performed according to the manufacturers’ instructions, as previously reported (35). To classify samples as positive, we applied the cut-off proposed by the manufacturers for the three commercial assays, while referring to the 99^th^ percentile for the in-house ELISA. Ninety-nine aPL negative serum samples (24 systemic lupus erythematosus patients and 75 healthy donors) were included to calculate assay-specific sensitivity and specificity at the manufacturers’ recommended cut-offs.

### Anti-β2GPI domain specificity

Anti-DI antibodies were measured by QUANTA Flash^®^ β2GPI Domain I CLIA (Inova Diagnostics, San Diego, CA, USA) (36), according to the manufacturer’s protocol and cut-off. Anti-β2GPI DIV/DV IgG were detected by in-house ELISA, with the positivity threshold set at titers >99^th^ percentile, as described (35). Reactivity against specific domains was measured by ELISA by using recombinant β2GPI wild-type (WT) and variants lacking domain I (ΔDI), domain II (ΔDII), domain III (ΔDIII), domain IV (ΔDIV) and domain V (ΔDV). Proteins were produced in HEK293 cells and used in ELISA as recently described (35, 37).

### Data analysis

Continuous data were presented as mean ± standard deviation or as median (25th–75th percentile), as appropriate. Categorical data were summarized as absolute and relative frequencies. Comparisons of continuous variables among anti-β2GPI IgG fine domain-specificity groups- reporting data as the optical density (OD) ratio of each single-domain lacking variant to the WT molecule - were performed using Kruskal-Wallis/Dunn’s adjusted multiple comparisons test. Proportions were compared using Chi-square test or Fisher’s exact test, as appropriate. All tests were two-sided and a p-value < 0.05 was considered statistically significant. All analyses were performed with GraphPad Prism 8 Software, Boston, US.

## Results

### Prevalence of anti-DI and anti-DIV/DV IgG antibodies in the study cohorts

**Figure 1:**
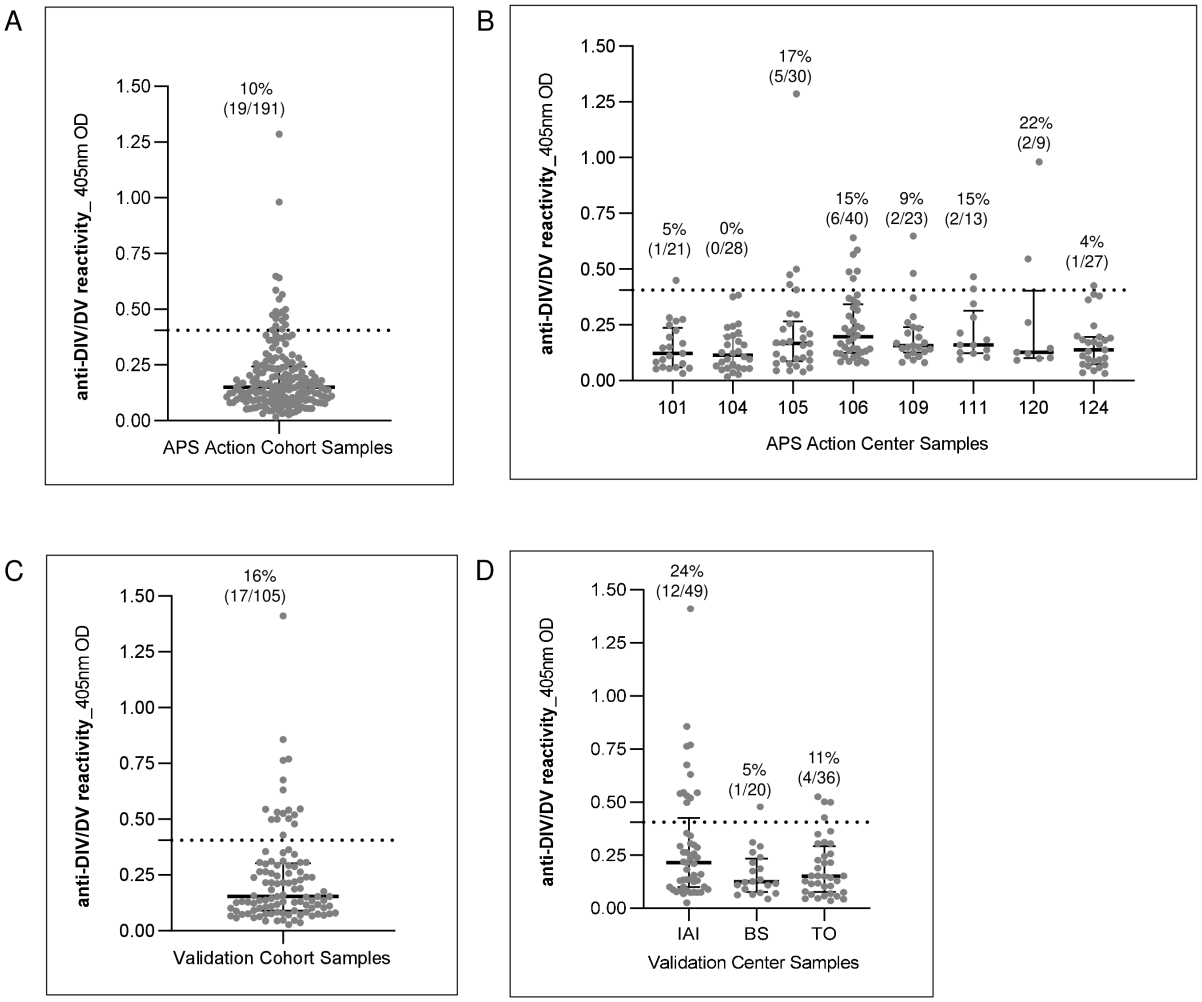
Anti-β2GPI DIV/DV specificity. Anti-DIV/DV IgG reactivity of: **(A)** APS Action sub-set of anti-β2GPI IgG positive patients (N = 191); **(B)** APS Action anti-β2GPI IgG positive patients grouped by recruiting center (eight centers; center ID: 101, 104, 105, 106, 109, 111, 120, 124); **(C)** validation cohort sub-set of anti-β2GPI IgG positive patients (N = 105); **(D)** validation cohort anti-β2GPI IgG positive patients grouped by recruiting center (three centers; center ID: IAI, BS, TO). Data are expressed as OD, optical density. Median with interquartile ranges is reported. Dotted line represents the in-house ELISA cut-off, and the number and percentage of positive samples are reported.

We first analyzed the APS ACTION discovery cohort to assess the prevalence of antibodies targeting DI and DIV/DV. Among the 291 enrolled patients, we focused on 191 individuals with confirmed anti-β2GPI IgG positivity, as validated by APS ACTION core laboratories by CLIA (**Table 1**). Of these, 149/191 (78%) met criteria for APS (33, 34), while 42/191 (22%) were non-APS classifiable aPL positive subjects. Anti-DI IgG antibodies (reacting with DI or DI-DIV/V) were detected in the majority of samples (147/191, 77%), reinforcing the notion that DI is a principal antigenic target in APS (**Table 1**). In contrast, anti-DIV/DV IgG antibodies (reacting with DIV/DV or DI-DIV/DV) were identified in a smaller subset (19/191, 10%) (**Table 1**, **Figure 1A**), with notable inter-center variability in detection rates (range: 0–22%) (**Figure 1B**). Monospecific anti-DIV/DV positivity (i.e., anti-DI negative) was distributed between non-classifiable and classified APS (2/42, 5% vs 4/149, 3%) (**Table 1**).

**Table 1:** Clinical, laboratory and demographic characteristics of the 191 anti-β2GPI IgG positive patients from the APS Action cohort.

Demographic features	No APS classification° (42/191, 22%)	APS^#^ (149/191, 78%)
Age (average ± SD)	54 ± 13	53 ± 13
Females	35/42 (83%)	100/149 (67%)
Males	7/42 (17%)	49/149 (33%)

Diagnosis	No APS Classification° (42/191, 22%)	APS^#^ (149/191, 78%)
PAPS (102/191, 53%)	/	102/149 (68%)
aPL without SARDs (22/191, 12%)	22/42 (52%)	/
SAPS (47/191, 25%)	/	47/149 (32%)
aPL with SARDs (20/191, 10%)	20/42 (48%)	/

aβ2GPI IgG domain specificity	No APS Classification° (42/191, 22%)	APS^#^ (149/191, 78%)
Single pos aDIV/DV (6/191, 3%)	2/42 (5%)	4/149 (3%)
Single pos aDI (134/191, 70%)	25/42 (59.5%)	109/149 (73%)
Double pos aDIV/DV, aDI (13/191, 7%)	4/42 (9.5%)	9/149 (6%)
Double neg aDIV/DV, aDI (38/191, 20%)	11/42 (26%)	27/149 (18%)

°NonAPS classifiable aPL positive subjects [34]; ^#^APS: 115/149 (77%) Thrombotic, 15/149 (10%) Obstetric, 19/149 (13%) Thrombotic and Obstetric; aPL, anti-phospholipid; APS, anti-phospholipid syndrome; PAPS, primary APS; SAPS, secondary APS; SARDs, Systemic Autoimmune Rheumatic Diseases; aβ2GPI, anti-β2-glycoprotein I; aDIV/DV, anti-β2GPI domain IV/V; aDI, anti-β2GPI domain I.

These findings were corroborated in an independent validation cohort. Among the 133 enrolled patients, we focused on 105 individuals with confirmed anti-β2GPI IgG positivity in at least one out of the four methods used (**Table 2**). Assay-specific sensitivity and specificity calculated at the recommended cut-offs are reported in **Supplementary Table 4** (**Table 4S**). Of these, 82/105 (78%) met criteria for APS (33, 34), while 23/105 (22%) were non-APS classifiable aPL positive subjects. Anti-DI IgG antibodies were detected in the majority of samples (67/105, 64%), and anti-DIV/DV IgG antibodies were identified in a smaller subset (17/105, 16%) (**Table 2**, **Figure 1C**), with notable inter-center variability in detection rates (range: 5–24%) (**Figure 1D**). Again, monospecific anti-DIV/DV positivity was distributed between non-classifiable and classified APS, but with higher difference in this cohort (4/23, 17% vs 5/82, 6%) (**Table 2**). We include a **Supplementary Table 5** (**Table 5S**) showing the characteristics of patients enrolled across units in the two cohorts.

**Table 2:** Clinical, laboratory and demographic characteristics of the 105 anti-β2GPI IgG positive patients from the validation cohort.

Demographic features	No APS classification ° (23/105, 22%)	APS^#^ (82/105, 78%)
Age (average ± SD)	44 ± 13	48 ± 10
Females	22/23 (96%)	61/82 (74%)
Males	1/23 (4%)	21/82 (26%)

Diagnosis	No APS classification ° (23/105, 22%)	APS^#^ (82/105, 78%)
PAPS (57/105, 54%)	/	58/82 (71%)
aPL without SARDs (11/105, 10%)	11/23 (48%)	/
SAPS (24/105, 23%)	/	24/82 (29%)
aPL with SARDs (12/105, 11%)	12/23 (52%)	/

aβ2GPI IgG domain specificity	No APS classification ° (23/105, 22%)	APS^#^ (82/105, 78%)
Single pos aDIV/DV (9/105, 9%)	4/23 (17%)	5/82 (6%)
Single pos aDI (59/105, 56%)	12/23 (52%)	47/82 (57%)
Double pos aDIV/DV, aDI (8/105, 8%)	0/23 (0%)	8/82 (10%)
Double neg aDIV/DV, aDI (29/105, 28%)	7/23 (31%)	22/82 (27%)

°Non-APS-classifiable aPL positive subjects [34]; ^#^APS: 52/82 (63%) Thrombotic, 13/82 (16%) Obstetric, 17/82 (21%) Thrombotic and Obstetric; aPL, anti-phospholipid; APS, anti-phospholipid syndrome; PAPS, primary APS; SAPS, secondary APS; SARDs, Systemic Autoimmune Rheumatic Diseases; aβ2GPI, anti-β2-glycoprotein I; aDIV/DV, anti-β2GPI domain IV/V; aDI, anti-β2GPI domain I.

### Anti-β2GPI positive samples reacting neither with DI nor with DIV/DV IgG

Anti-β2GPI positive samples reacting neither with DI nor with DIV/DVIgG binding against the whole β2GPI cannot be explained by the reactivity against DI and DIV/DV only. In fact, in the APS Action cohort 38 out of 191 (20%) anti-β2GPI IgG positive samples were negative for both anti-DI and anti-DIV/DV antibodies. Of these, 27/38 (71%) were classified as APS, and 11/38 (29%) were non-classifiable APS (27 out of the 149-total classified as APS (18%) and 11 out of the 42 total non-classifiable APS (26%) subjects in **Table 1**).

Comparable results were found also in the validation cohort. In fact, 29 out of 105 (28%) anti-β2GPI IgG positive samples were negative for both anti-DI and anti-DIV/DV. Of these, 22/29 (76%) were classified as APS, and 7/29 (24%) were non-classifiable APS (22 out of the 82-total classified as APS (27%) and 7 out of the 23 total non-classifiable APS (31%) subjects in **Table 2**).

### Anti-β2GPI IgG single domain-specificity in the validation cohort samples

The lack of reactivity against DI and DIV/DV in samples positive for anti-β2GPI (whole molecule) IgG raises the issue whether the binding can be sustained by antibodies against other domains. To assess the β2GPI single-domain IgG reactivity, the next step was to characterize the binding to single domain-lacking constructs. To do this, we selected 44 medium/high (40–79 AU medium, ≥ 80 AU high) positive sera by in-house β2GPI ELISA from the validation cohort. The 44−sample subset clinical characteristics were comparable to those of the overall 105-Italian cohort (**Supplementary Table 6**).

**Figure 2:**
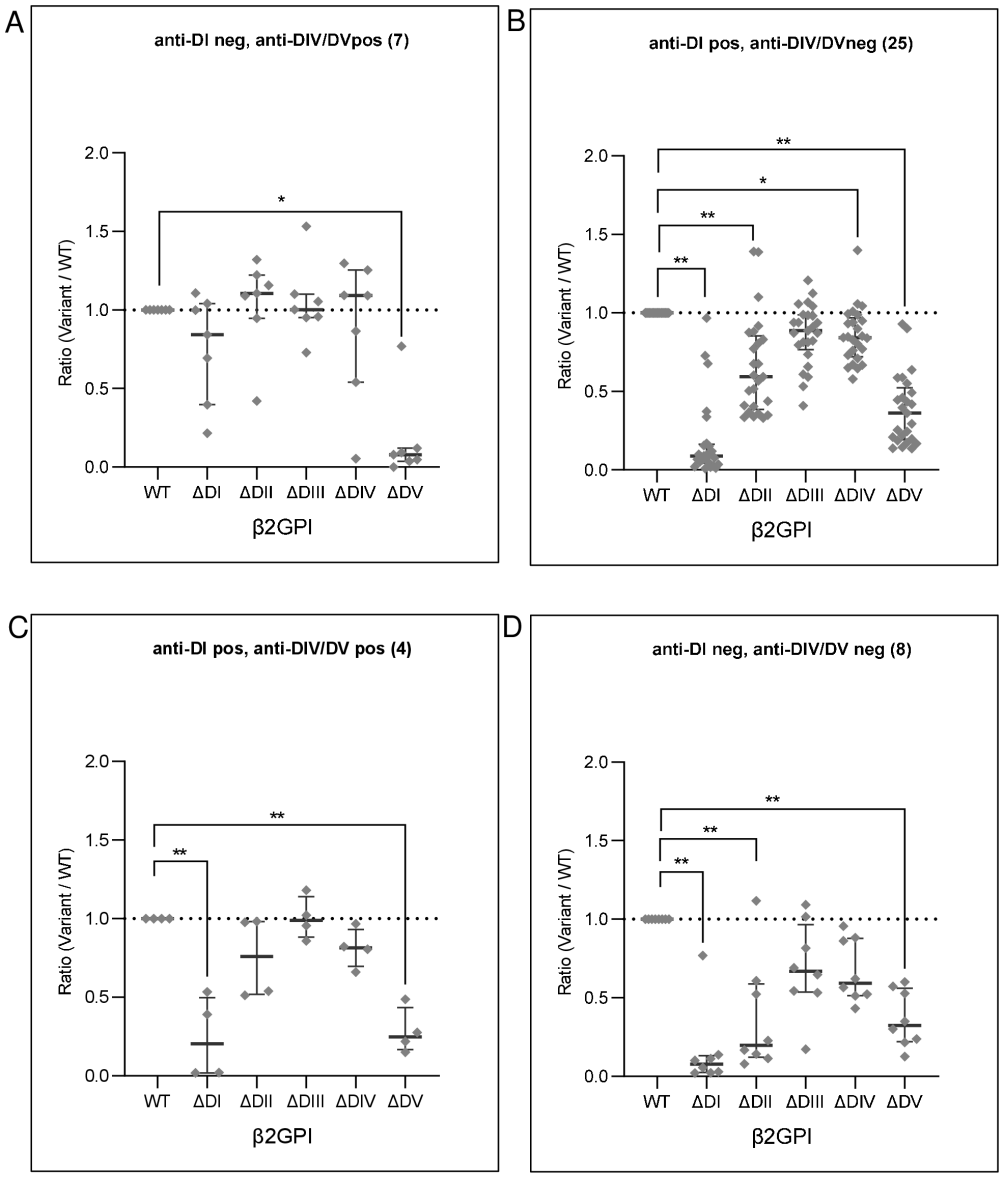
Reactivity against single domain-deleted β2GPI variants of anti-DI and/or DIV/DV positive patients. Forty-four anti-β2GPI IgG positive samples were grouped according to their DI and/or DIV/DV specificity. The graphs show reactivities (OD405nm) against the single domain-deleted variants (ΔDI, ΔDII, ΔDIII, ΔDIV, ΔDV) of **(A)** anti-DIV/DV monospecific positive samples [N = 7; 3/7 non-APS classifiable aPL positive subjects (not classified APS), 4/7 APS (34)]; **(B)** anti-DI monospecific positive samples (N = 25; 8/25 not classified APS, 17/25 APS); **(C)** anti-DI, DIV/DV double positive samples (N = 4; 4/4 APS); **(D)** anti-DI, DIV/DV double negative samples (N = 8; 1/8 not classified APS, 7/8 APS). Values are expressed as ratio to the whole molecule (WT). Median with interquartile ranges is reported, p-values calculated with Kruskal-Wallis/Dunn’s adjusted multiple comparisons test **p< 0.001, *p<0.05.

**Figure 2** shows the reactivity patterns of these samples grouped as anti-DIV/DV single positive (i.e., anti-DI negative) (7/44; **Figure 2A**), anti-DI single positives (i.e., anti-DIV/DV negative) (25/44; **Figure 2B**), positive for both anti-DI and anti-DIV/DV (4/44; **Figure 2C**), and as negative for both anti-DI and anti-DIV/DV (8/44; **Figure 2D**). The clinical classification of samples is reported in the Figure legend for each of the subsets.

As expected, anti-DIV/DV monospecific samples lost their reactivity towards the ΔDV almost completely (**Figure 2A**). Conversely, a statistically significant DI-dependent reduction of reactivity was evidenced in anti-DI positive sera (both monospecific and associated with anti-DIV/DV activity) (**Figures 2B, C**). Both anti-DI positive sub-groups displayed a considerable reduction of their binding to ΔDV (**Figures 2B, C**), and monospecific anti-DI also showed binding reduction to ΔDII, and to ΔDIV (**Figure 2B**).

Samples negative for both anti-DI and anti-DIV/DV displayed a significant decrease of reactivity against ΔDI or ΔDII, or ΔDV (**Figure 2D**).

The reactivity reduction towards ΔDIII was not significant in all the sub-groups (**Figures 2A–D**).

**Figure 3:**
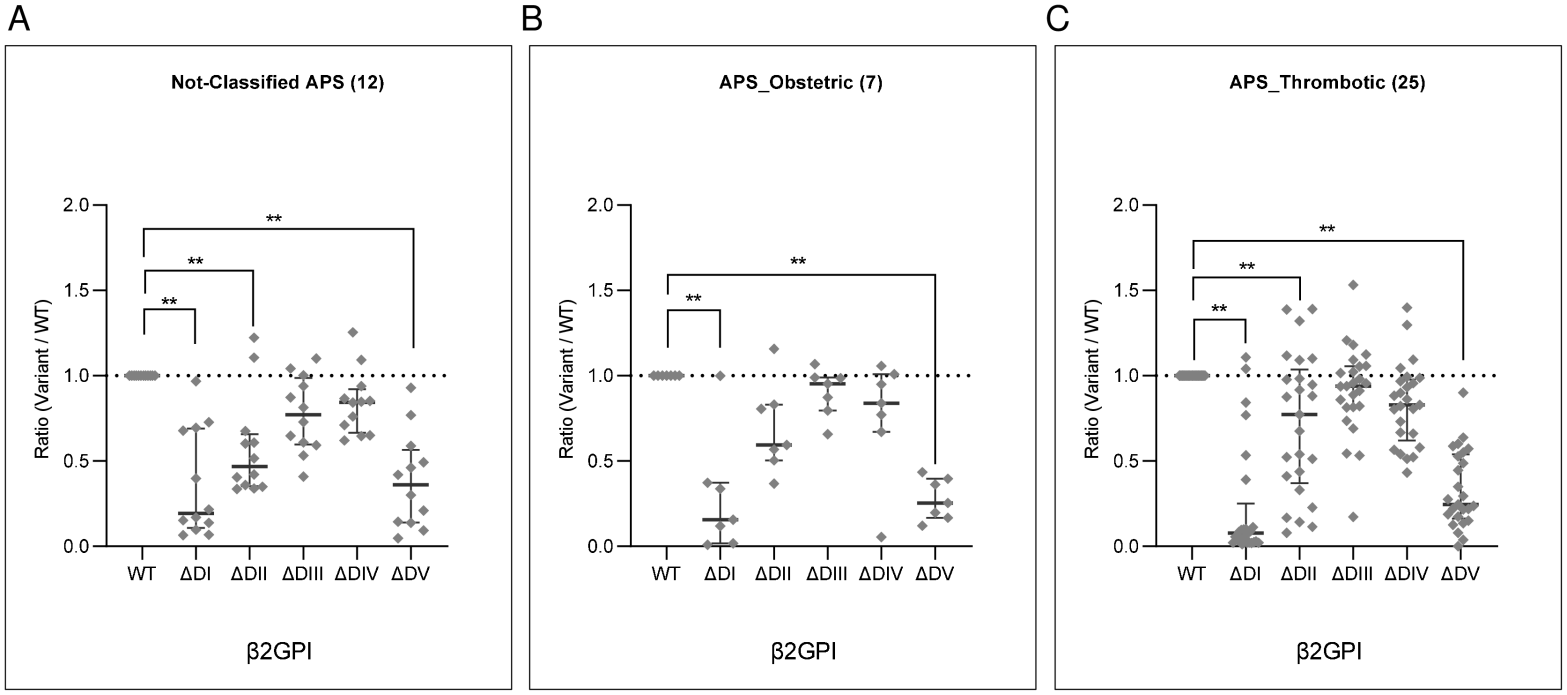
Reactivity against single domain-deleted β2GPI variants of non-APS classifiable aPL positive subjects and APS patients. Forty-four anti-β2GPI IgG positive samples were grouped according to their diagnosis. The graphs show reactivities (OD405nm) against the single domain-deleted variants (ΔDI, ΔDII, ΔDIII, ΔDIV, ΔDV) of **(A)** 12/44 non-APS classifiable aPL positive subjects, and 32/44 APS patients (34), **(B)** 7/44 with only obstetric events, and **(C)** 25/44 with thrombotic with or without obstetric events. Values are expressed as ratio to the whole molecule (WT). Median with interquartile ranges is reported, p-values calculated with Kruskal-Wallis/Dunn’s adjusted multiple comparisons test **p< 0.001.

Anti-β2GPI DI, DII and DV-dependent reactivity was observed both in classifiable and non-classifiable APS (**Figures 3A–C**). The pure obstetric APS subgroup (7/32) didn’t show a significant reduction towards the variant lacking DII (**Figure 3B**).

### Domain specificity is associated with discordant results in anti-β2GPI assays

As detailed in the Methods section, we measured anti-β2GPI IgG in the validation cohort using four different techniques (in-house and commercial ELISA, CLIA and FEIA), and found that 28/133 were concordantly negative in all the four methods, whilst 105/133 samples were positive for at least one method. Among these 105 anti-β2GPI IgG positive samples, 54 were concordant (i.e., positive in all the four assays), while 51 were discordant (i.e., positive in at least one and negative in at least one assay). The discordant patients displayed the highest presence of anti-DIV/DV single-positives and anti-DI/anti-DIV/DV double-negatives, compared to the concordant ones: anti-DIV/DV single-positives 16% vs 2%, and anti-DI/anti-DIV/DV double-negatives 45% vs 11%. Conversely, the concordant positive subset displayed higher frequency of monospecific anti-β2GPI DI compared to discordant samples (78% vs 33%; Chi-square test, df 3, N = 105, p<0.0001, **Table 3**). The non-classifiable APS (N = 23) were 14/23 (60%) discordant and 9/23 (40%) concordant, conversely the classified APS (N = 82) were 37/82 (45%) discordant and 45/82 (55%) concordant (**Table 3**). **Supplementary Table 3** (**Table 3S**) highlights how anti-DIV/DV single positive and anti-DI-DIV/DV double negative discordant samples were detected by the four anti-β2GPI IgG assays.

**Table 3:** Frequencies of discordant/concordant, not-classified/classified samples in the anti-β2GPI IgG positive validation cohort.

Positives at least one method° (N = 105)	Discordant^#^ (N = 51)	Concordant^#^ (N = 54)	P-value^§^
Single pos aDIV/DV	8 (16%)	1 (2%)	<.0001
Single pos aDI	17 (33%)	42 (78%)	
Double pos aDIV/DV, aDI	3 (6%)	5 (9%)	
Double neg aDIV/DV, aDI	23 (45%)	6 (11%)	

Positives at least one method and No APS classification^Δ^ (N = 23)	Discordant (N = 14)	Concordant (N = 9)	
Single pos aDIV/DV	4 (29%)	0 (0%)	0.0217
Single pos aDI	4 (29%)	8 (89%)	
Double pos aDIV/DV, aDI	0 (0%)	0 (0%)	
Double neg aDIV/DV, aDI	6 (43%)	1 (11%)	

Positives at least one method and Yes APS classification (N = 82)	Discordant (N = 37)	Concordant (N = 45)	
Single pos aDIV/DV	4 (11%)	1 (2%)	0.0002
Single pos aDI	13 (35%)	34 (76%)	
Double pos aDIV/DV, aDI	3 (8%)	5 (11%)	
Double neg aDIV/DV, aDI	17 (46%)	5 (11%)	

°Samples positives in at least one out of the four anti-β2-glycoprotein I IgG methods. ^#^Concordant samples were positive in all the four assays, discordant were positive in at least one and negative in at least one assay; ^Δ^Non-APS-classifiable aPL positive subjects [34]; aβ2GPI, anti-β2-glycoprotein I; aDIV/DV, anti-domain V; aDI, anti-domain I; ^§^*χ*^2^(df =3; N = 105, N = 23, N = 82).

**Figure 4:**
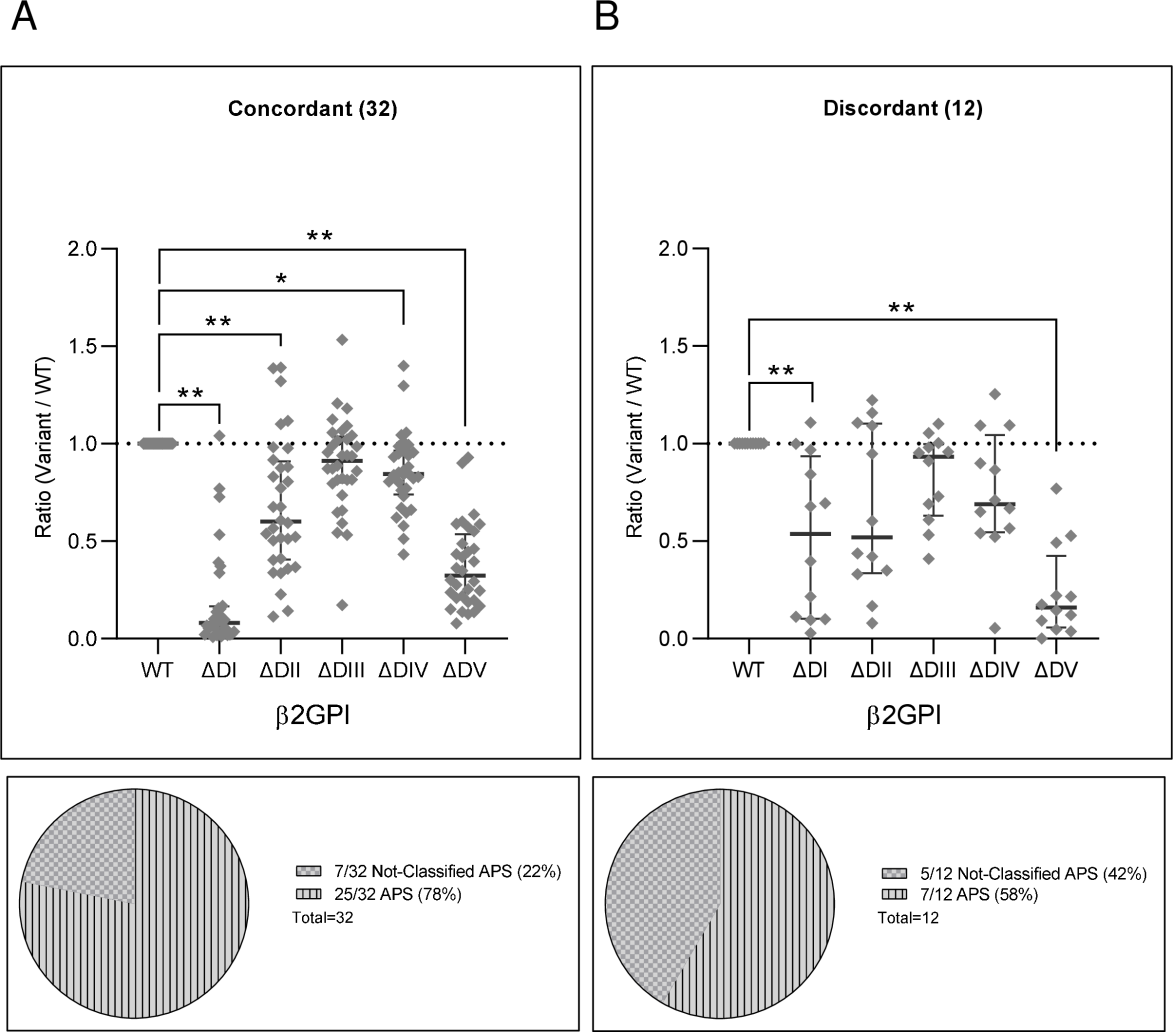
Domain-dependent anti-β2GPI reactivity of concordant or discordant samples in the different anti-β2GPI IgG assays. Forty-four anti-β2GPI IgG positive samples were grouped according to **(A)** their four-assay concordance (N = 32) or **(B)** discordance (N = 12). The graphs show their reactivities (OD405nm) against the single domain-deleted variants (ΔDI, ΔDII, ΔDIII, ΔDIV, ΔDV). Values are expressed as ratio to the whole molecule (WT). Pie charts describe the proportion of non-APS classifiable aPL positive subjects, and APS patients (34) in the concordant and discordant groups. Median with interquartile ranges is reported, p-values calculated with Kruskal-Wallis/Dunn’s adjusted multiple comparisons test **p< 0.001, *p<0.05.

Among the 44 validation cohort patients analyzed for the reactivity against domain-deleted constructs, 32 out of the total 54 concordant and 12 out of the 51 discordant samples met the medium/high inclusion criteria for the assay (**Figure 4**; **Supplementary Table 7**). Concordant samples displayed significant DI-, DII-, DIV- and DV-dependency (**Figure 4A**), whereas discordant samples DI- and DV- dependency only (**Figure 4B**). Concordant samples had higher frequency of APS samples than discordant ones (Pie Charts in **Figures 4A, B**): specifically, concordant samples were 25/32 (78%) APS and 7/32 (22%) not-classifiable APS, whereas discordant samples were 7/12 (56%) APS and 5/12 (42%) not-classifiable APS.

## Discussion

The present study expands our knowledge on the domain specificity of anti-β2GPI IgG in two large, multicenter cohorts of aPL-positive samples, confirming their heterogeneity and suggesting that distinct reactivity patterns may explain, at least in part, the discordance among diagnostic assays.

Autoimmune response against the triggering antigen can be polyclonal and directed against different epitopes. Since not all autoantibodies may display the same pathogenic/diagnostic value, the antigen specificity characterization is helpful for better understanding the disease pathogenesis and improving diagnosis.

We investigated two different cohorts of aPL-positive samples representative of the patient population in real life. The first cohort included patients enrolled by APS ACTION international network (33, 34), and the second one by an Italian multicenter network (35). The anti-domain β2GPI IgG specificity was characterized by combining direct assays to detect anti-DI and anti-DIV/DV reactivity and indirect assays that investigated IgG binding to five selectively single-domain lacking β2GPI molecules.

The emerging primary data confirmed the crucial role of DI and also highlighted the additive involvement of DV as antibody targets. The single as well as the double anti-DI and anti-DIV/DV positivity by the direct assays emerged as the prevalent and stronger reactivity in classifiable APS of both the APS ACTION and the validation cohort. The substantial binding loss to DI-deleted constructs in most β2GPI IgG positive samples by the indirect assay further supports the main role of DI.

Anti-DIV/DV monospecific positive samples exist in both cohorts. This is a less frequent condition already observed in the aPL-positive population, mainly associated to non-classifiable APS by our group and others in relatively small cohorts (26–29). We previously showed that anti-DIV/DV β2GPI IgG were mainly directed against DV, and failed to induce thrombosis in a rat model of vascular APS (37). The loss of reactivity of anti-DIV/DV monospecific positive samples towards ΔDV molecule only is consistent with such specificity. The relationship between these antibodies and the clinical outcome in APS patients appears to be controversial in the two cohorts: anti-DIV/DV mono-specificity in not-classifiable compared to classifiable APS was only slightly higher in the APS ACTION’s cohort, and frankly higher in the validation one. Even though preliminary data support the non-pathogenic role of anti-DIV/DV antibodies, their clinical value remains an open issue (26–29, 37). Anti-β2GPI IgG positive samples negative for anti-DI and anti-DIV/DV antibodies were found in both cohorts, suggesting that they may recognize other domains as targets. In line with this, these sera showed a significant reduction in reactivity towards the β2GPI molecule, which selectively lacks the DII. Unexpectedly, despite the absence of direct reactivity by the anti-DI and DIV/DV assays available, these samples also showed a significant decrease in reactivity towards the β2GPI molecules, which selectively lack DI or DV. towards The loss of antibody reactivity upon deletion of DII, was observed also in monospecific anti-DI positive samples. Although the origin of this reactivity remains unclear, recent studies suggest heterogeneity within anti-DI antibodies (38), with some antibodies recognizing epitopes confined to DI and others potentially extending into DII. Alternatively, domain deletions may result in structural changes that obscure pre-existing epitopes. These possibilities warrant further investigation.

We observed that the anti-DI-positive sera negative for anti-DIV/DV lost their reactivity upon DV deletion too, and in general DV deletion was associated with a decreased reactivity in all the anti-β2GPI IgG positive samples. Given that both DI and DV are positively charged (2, 4, 5, 39), and that anti-DI antibodies, though recognizing a conformational epitope, are low-affinity and electrostatically driven (38–40), some degree of cross-reactivity may occur.

Domain III deletion didn’t affect any serum sample reactivity, and this is consistent with it being a high glycosylation site (2).

The availability of anti-β2GPI IgG values by four distinct detection methods (35), combined with direct and indirect domain-specific assays, enabled us to evaluate concordance across the four diagnostic platforms from the β2GPI-domain perspective. Monospecific anti-DIV/DV-positive and double-negative anti-DI/anti-DIV/DV samples gave more frequently discordant positive results, whereas anti-DI-positive samples showed higher concordance. In our previous work (35), we observed that despite efforts to reduce inter-assay discrepancies through cut-off adjustment, complete harmonization remains difficult. The two assays that benefited most from the polyclonal reference material–based harmonization method likely share similarities in antigen source and preparation (35). Overall, these data suggest that current diagnostic assays might differ in their sensitivity to β2GPI domains – particularly non-DI epitopes – likely due to differences in antigen preparation, binding to solid phase supports, and epitope exposure, in addition to assay specific chemical characteristics. In this regard, we previously showed that monospecific anti-DIV/DV IgG tested positive in β2GPI (whole molecule) but not in β2GPI-dependent aCL assay. Likely the discrepancy can be due to IgG inability in recognizing DV engaged in CL-coated plates binding (37). From a practical standpoint, this means that for a minor but existing group of aPL positive subjects the specific choice of the anti-β2GPI IgG assay matters, and employing multiple anti-β2GPI IgG assays may improve their diagnostic accuracy.

In conclusion, our findings document a heterogeneous response of IgG anti-β2GPI antibodies in APS, with DI as the dominant target, but also involving mainly DII and DV, and, to a lesser extent, DIV. This supports and justifies the use of polyclonal anti-β2GPI IgG reference materials instead of monoclonal antibodies. In this regard, a recently validated polyclonal reference material, ERM^®^-DA477/IFCC, displays a similar domain polyreactivity (35). Finally, this domain-dependent heterogeneity may help to understand the discordant results observed in the current diagnostic assays and underscores the need for more research into epitope definition to support the development of more precise serological tools for APS diagnostics.

## Data Availability

APS ACTION discovery cohort dataset is available upon reasonable request to the APS ACTION committee (ErkanD@HSS.EDU). Validation cohort dataset is available at the Zenodo repository link https://doi.org/10.5281/zenodo.15755268, upon reasonable request to the corresponding author.
