# Correction: Anti-β2GPI IgG display a broad reactivity against different β2GPI domains beyond domain 1: results from the APS ACTION and multi-center Italian cohorts

**DOI:** 10.3389/fimmu.2026.1879170

**Published:** 2026-05-26

**Authors:** Claudia Grossi, Caterina Bodio, Suresh Kumar, Elisa Bison, Ricard Cervera, Maria Gerosa, Michael Mahler, Diana Paredes-Ruiz, Silvia Piantoni, Massimo Radin, Savino Sciascia, Esther Rodriguez-Almaraz, Maria G. Tektonidou, Angela Tincani, Vittorio Pengo, Davide Soranna, Antonella Zambon, Maria Laura Bertolaccini, Hannah Cohen, Doruk Erkan, Nicola Pozzi, Francesco Tedesco, Maria Orietta Borghi, Pier Luigi Meroni

**Affiliations:** 1 Laboratory of Immuno-Rheumatology, IRCCS Istituto Auxologico Italiano, Milan, Italy; 2 Edward A. Doisy Department of Biochemistry and Molecular Biology, Saint Louis University School of Medicine, St. Louis, MO, United States; 3 Thrombosis Research Laboratory, Department of Cardio-Thoracic-Vascular Sciences and Public Health, University of Padova, Padova, Italy; 4 Department of Autoimmune Diseases, Hospital Clinic, University of Barcelona, Barcelona, Spain; 5 Dipartimento di Scienze Cliniche e di Comunità, Dipartimento di Eccellenza 2023-2027, University of Milan, Milan, Italy; 6 Rheumatology Clinic, ASST-G.Pini-CTO, Milan, Italy; 7 Research and Development, Headquarters & Technology Center Autoimmunity, Werfen, San Diego, CA, United States; 8 Autoimmune Diseases Research Unit, Department of Internal Medicine, Biobizkaia Health Research Institute, Hospital Universitario, Cruces, Spain; 9 Rheumatology and Clinical Immunology Unit, ASST Spedali Civili, Department of Clinical and Experimental Sciences, University of Brescia, ERN-Reconnect Member, Brescia, Italy; 10 Department of Clinical and Biological Sciences, University Center of Excellence on Nephrologic, Rheumatologic and Rare Diseases (ERK-Net, ERN-Reconnect and RITAERN Member) with Nephrology and Dialysis Unit and Center of Immuno- Rheumatology and Rare Diseases (CMID), Coordinating Center of the Interregional Network for Rare Diseases of Piedmont and Aosta Valley, San Giovanni Bosco Hub Hospital, University of Turin, Turin, Italy; 11 Hospital Universitario 12 de Octubre, Madrid, Spain; 12 Rheumatology Unit, First Department of Propaedeutic Internal Medicine, Joint Academic Rheumatology Program, Medical School, National and Kapodistrian University of Athens, Athens, Greece; 13 Biostatistic Unit, IRCCS Istituto Auxologico Italiano, Milan, Italy; 14 Laboratory of Quantitative Methods for Life, Health and Society, Department of Statistics and Quantitative Methods, University of Milano-Bicocca, Milan, Italy; 15 Academic Department of Vascular Surgery, School of Cardiovascular and Metabolic Medicine & Sciences, King’s College, London, United Kingdom; 16 Department of Haematology, University College London, London, United Kingdom; 17 Barbara Volcker Center for Women and Rheumatic Diseases, Hospital for Special Surgery, Weill Cornell Medicine, New York, NY, United States

**Keywords:** antibodies, antiphospholipid syndrome, diagnostic assays, domains, β2GPI

Affiliation “5 Dipartimento di Scienze Cliniche e di Comunità, Dipartimento di Eccellenza 2023-2027, University of Milan, Milan, Italy” was erroneously given as “5 Dipartimento di Scienze Cliniche e di Comunità, University of Milan, Milan, Italy” and “6 Dipartimento di Eccellenza, University of Milan, Milan, Italy”.

Affiliation “Department of Clinical and Biological Sciences, University Center of Excellence on Nephrologic, Rheumatologic and Rare Diseases (ERK-Net, ERN-Reconnect and RITA-ERN Member) with Nephrology and Dialysis Unit and Center of Immuno-Rheumatology and Rare Diseases (CMID), Coordinating Center of the Interregional Network for Rare Diseases of Piedmont and Aosta Valley, San Giovanni Bosco Hub Hospital, University of Turin, Turin, Italy” was omitted for author “Savino Sciascia”.

Author “Savino Sciascia” was erroneously assigned to affiliation “Hospital Universitario 12 de Octubre, Madrid, Spain”.

The original version of this article has been updated.

