# Supplementary material for: Anti-β2GPI IgG display a broad reactivity against different β2GPI domains beyond domain 1: results from the APS ACTION and multi-center Italian cohorts
Source: Front Immunol. 2026 May 1;17:1809192. doi: 10.3389/fimmu.2026.1809192 (PMC13176316; doi:10.3389/fimmu.2026.1809192)
Supplement: Supplementary file 1 [file SupplementaryFile1.pdf]

Table 1S. Clinical, Laboratory and Demographic characteristics of the 291 aPL positive subjects from the APS ACTION cohort

**Demographic features**	No APS Classification° (78/291, 27%)	APS$^{\infty}$ (213/291, 73%)
Age (average±SD)	54±13	55±13
Females	57/78 (73%)	145/213 (68%)
Males	21/78 (27%)	68/213 (32%)
Diagnosis		
PAPS (149/291, 51%)	/	149/213 (70%)
aPL without SARDs (42/291, 14%)	42/78 (54%)	/
SAPS (64/291, 22%)	/	64/213 (30%)
aPL with SARDs (36/291, 12%)	36/78 (46%)	/
**Laboratory features**		
Criteria aPL History[#]		
Positive aβ2GPI IgG	42/78 (54%)	149/213 (70%)
Positive aβ2GPI IgM	15/78 (19%)	49/213 (23%)
Positive aCL IgG	36/78 (46%)	138/213 (65%)
Positive aCL IgM	20/78 (26%)	56/213 (26%)
Positive LA	51/78 (65%)	163/213 (76%)
**aβ2GPI IgG Domain Specificity**[ǂ]		
Single pos aDIV/DV	5/78 (6%)	7/213 (3%)
Single pos aDI	26/78 (33%)	113/213 (53%)
Double pos aDIV/DV, aDI	4/78 (5%)	10/213 (5%)
Double neg aDIV/DV, aDI	43/78 (55%)	83/213 (39%)

°Non-APS-classifiable aPL positive subjects [34]; $^{\infty}$APS: 165/213 (78%) Thrombotic, 24/213 (11%) Obstetric, 24/213 (11%) Thrombotic and Obstetric; [#]Criteria aPL history was available from APS ACTION's dataset; [ǂ]aβ2GPI IgG domain specificity evaluated in this study; aPL, anti-phospholipid; APS, anti-phospholipid syndrome; PAPS, primary APS; SAPS, secondary APS; aPL without or with SARDs, aPL positive subjects without or with Systemic Autoimmune Rheumatic Diseases; aβ2GPI, anti-β2-glycoprotein I; aCL, anti-cardiolipin; LA, lupus anticoagulant; aDIV/DV, anti-β2GPI domain IV/V; aDI, anti-β2GPI domain I

Table 2S. Clinical, Laboratory and Demographic characteristics of the 133 aPL positive subjects for the validation cohort

**Demographic features**	No APS Classification° (34/133, 26%)	APS$^{\infty}$ (99/133, 74%)
Age (average±SD)	47±12	49±12
Females	30/34 (88%)	74/99 (75%)
Males	4/34 (12%)	25/99 (25%)
**Diagnosis**		
PAPS (64/133, 48%)	/	64/99 (65%)
aPL without SARDs (16/133, 12%)	16/34 (47%)	/
SAPS (35/133, 26%)	/	35/99 (35%)
aPL with SARDs (18/133, 14%)	18/34 (53%)	/
**Laboratory Features**		
Criteria aPL History[#]		
Positive aβ2GPI IgG	17/34 (50%)	63/99 (63%)
Positive aβ2GPI IgM	15/34 (44%)	25/99 (25%)
Positive aCL IgG	15/34 (44%)	65/99 (66%)
Positive aCL IgM	13/34 (38%)	26/99 (26%)
Positive LA	25/34 (73%)	85/99 (86%)
**aβ2GPI IgG Domain Specificity**[ǂ]		
Single pos aDIV/DV	4/34 (12%)	5/99 (5%)
Single pos aDI	13/34 (38%)	48/99 (48%)
Double pos aDIV/DV, aDI	0/34 (0%)	8/99 (8%)
Double neg aDIV/DV, aDI	17/34 (50%)	38/99 (38%)

°Non-APS-classifiable aPL positive subjects [34]; $^{\infty}$APS: 67/99 (68%) Thrombotic, 14/99 (14%) Obstetric, 18/99 (18%) Thrombotic and Obstetric; [#]Criteria aPL history refers to ELISA-based information from each recruiting center; [ǂ]aβ2GPI IgG domain specificity evaluated in this study; aPL, anti-phospholipid; APS, anti-phospholipid syndrome; PAPS, primary APS; SAPS, secondary APS; aPL without or with SARDs, aPL positive subjects without or with Systemic Autoimmune Rheumatic Diseases; aβ2GPI, anti-β2-glycoprotein I; aCL, anti-cardiolipin; LA, lupus anticoagulant; aDIV/DV, anti-β2GPI domain IV/V; aDI, anti-β2GPI domain I

Table 3S. Anti-DIV/DV single positive and anti-DI-DIV/DV double negative discordant samples: distribution of negative and positive results among the four anti-β2GPI IgG assays

Method	anti-DIV/DV single positives (8/51)	
	Negatives	Positives
in-house ELISA	2/8 (25%)	6/8 (75%)
QUANTA Lite® β2GPI IgG ELISA	3/8 (38%)	5/8 (63%)
EliA™ β2-Glycoprotein I IgG FEIA	3/8 (38%)	5/8 (63%)
QUANTA Flash® β2GPI IgG CLIA	4/8 (50%)	4/8 (50%)
	anti-DI-DIV/DV double negatives (23/51)	
	Negatives	Positives
in-house ELISA	19/23 (83%)	4/23 (17%)
QUANTA Lite® β2GPI IgG ELISA	22/23 (96%)	1/23 (4%)
EliA™ β2-Glycoprotein I IgG FEIA	23/23 (100%)	0/23 (0%)
QUANTA Flash® β2GPI IgG CLIA	1/23 (4%)	22/23 (96%)

Table 4S. Sensitivity-specificity of the four anti-β2GPI IgG detecting methods. Method sensitivity and sensibility were evaluated using the 133 aPL positive validation cohort, and 75 healthy donors/24 SLE aPL negative controls

**Assay name (product code)**	**Manufacturer/ developer**	**Sensitivity***	**Specificity***
In-house β2GPI ELISA IgG ELISA	IAI-BS Labs	58% (49% to 67%)	100% (96% to 100%)
QUANTA Lite® β2GPI IgG (708665)	Inova Diagnostics	47% (38% to 55%)	99% (94% to 100%)
EliA™ β2-Glycoprotein I IgG	Phadia, Thermo Fisher Scientific	47% (38% to 55%)	100% (96% to 100%)
QUANTA Flash® β2GPI IgG (701248)	Inova Diagnostics	75% (66% to 82%)	94% (86% to 98%)

* Based on manufacturer-recommended method cut-off

Table 5S. Single-center diagnosis distribution in the two anti-β2GPI IgG positive cohorts of the study

APS ACTION 191 cohort									
	Centers								
	**101 (N=21)**	**104 (N=28)**	**105 (N=30)**	**106 (N=40)**	**109 (N=23)**	**111 (N=13)**	**120 (N=9)**	**124 (N=27)**	**p-value §**
**Diagnosis**									
**PAPS**	12 (57%)	14 (50%)	18 (60%)	25 (63%)	13 (57%)	7 (54%)	1 (11%)	12 (44%)	
**SAPS**	4 (19%)	8 (29%)	5 (17%)	12 (30%)	3 (13%)	2 (15%)	3 (33%)	10 (37%)	< 0.0001
**aPL_SARDs**	1 (5%)	3 (11%)	0 (0%)	3 (8%)	2 (9%)	3 (23%)	4 (44%)	4 (15%)	
**aPL_noSARDs**	4 (19%)	3 (11%)	7 (23%)	0 (0%)	5 (22%)	1 (8%)	1 (11%)	1 (4%)	

Italian 105 cohort				
	Centers			
	**IAI (N=49)**	**BS (N=20)**	**TO (N=36)**	**p-value°**
**PAPS**	31 (63%)	11 (55%)	16 (44%)	
**SAPS**	9 (18%)	3 (15%)	12 (33%)	< 0.0001
**aPL_SARDs**	0 (0%)	6 (30%)	6 (17%)	
**aPL_noSARDs**	9 (18%)	0 (0%)	2 (6%)	

PAPS, primary APS; SAPS, secondary APS; aPL+, aPL positive subjects without Systemic Autoimmune Rheumatic Diseases; aPL_SARDs, aPL positive subjects with Systemic Autoimmune Rheumatic Diseases; §χ^2(df =21; N =191); °χ^2(df =6; N =105)

Table 6S. Clinical characteristic frequencies in the total 105-anti-β2GPI IgG positive validation cohort and in its 44-samples subset analyzed with the single-domain-deleted constructs

**Diagnosis**	**Total (N = 105)**	**Subset (N = 44)**
PAPS	58 (55%)	25 (57%)
SAPS	24 (23%)	7 (16%)
aPL+	11 (10%)	6 (14%)
aPL_SARDs	12 (11%)	6 (14%)
**Classification**		
Thrombotic	52 (50%)	16 (36%)
Obstetric	13 (12%)	7 (16%)
Thrombotic and Obstetric	17 (16%)	9 (20%)
aPL+ and aPL_SARDs	23 (22%)	12 (27%)

PAPS, primary APS; SAPS, secondary APS; aPL+, aPL positive subjects without Systemic Autoimmune Rheumatic Diseases; aPL_SARDs, aPL positive subjects with Systemic Autoimmune Rheumatic Diseases

Table 7S. Discordant/concordant sample and domain-specificity frequencies in the 105-anti-β2GPI IgG positive validation cohort and in its 44-samples subset analyzed with the single-domain-deleted constructs

**aβ2GPI IgG Positivity Concordance**	**Total (N = 105)**	**Subset (N = 44)**	
Discordant Positives	51 (49%)	12 (27%)	
Concordant Positives	54 (51%)	32 (73%)	
**Positives at least one method° (N= 105)**	**Discordant# (N = 51)**	**Concordant# (N = 54)**	**p-value §**
Single pos aDIV/DV	8 (16%)	1 (2%)	< 0.0001
Single pos aDI	17 (33%)	42 (78%)	
Double pos aDIV/DV, aDI	3 (6%)	5 (9%)	
Double neg aDIV/DV, aDI	23 (45%)	6 (11%)	
**Single domain deleted construct analyzed samples△ (N= 44)**	**Discordant (N = 12)**	**Concordant (N = 32)**	**p-value°**
Single pos aDIV/DV	6 (50%)	1 (3%)	< 0.0001
Single pos aDI	4 (33%)	21 (66%)	
Double pos aDIV/DV, aDI	0 (0%)	4 (13%)	
Double neg aDIV/DV, aDI	2 (17%)	6 (19%)	

°Samples positives in at least one out of the four anti-β2-glycoprotein I IgG methods. #Concordant samples were positive in all the four assays, discordant were positive in at least one and negative in at least one assay; △Subset of samples analyzed by single domain deleted constructs; aβ2GPI, anti-β2-glycoprotein I; aDIV/DV, anti-β2GPI domain IV/V; aDI, anti-domain I; §χ^2(df =3; N =105); °χ^2(df =3; N =44)
